# Storage of bronchoalveolar lavage fluid and accuracy of microbiologic diagnostics in the ICU: a prospective observational study

**DOI:** 10.1186/cc12814

**Published:** 2013-07-11

**Authors:** Nikolaus Kneidinger, Joanna Warszawska, Peter Schenk, Valentin Fuhrmann, Andja Bojic, Alexander Hirschl, Harald Herkner, Christian Madl, Athanasios Makristathis

**Affiliations:** 1 Department of Internal Medicine III, Intensive Care Unit 13H1, Division of Gastroenterology and Hepatology, Medical University of Vienna, Austria; 2 Department of Internal Medicine V, Ludwig Maximilian University, Munich, Germany; 3 Department of Pulmonology, Landesklinikum Hochegg, Austria; 4 Department of Internal Medicine I, Intensive Care Unit 13I2, Medical University of Vienna, Austria; 5 Department of Laboratory Medicine, Division of Clinical Microbiology, Medical University Vienna, Austria; 6 Department of Emergency Medicine, Medical University of Vienna, Austria

## Abstract

**Introduction:**

Early initiation of appropriate antimicrobial treatment is a cornerstone in managing pneumonia. Because microbiologic processing may not be available around the clock, optimal storage of specimens is essential for accurate microbiologic identification of pathogenetic bacteria. The aim of our study was to determine the accuracy of two commonly used storage approaches for delayed processing of bronchoalveolar lavage in critically ill patients with suspected pneumonia.

**Methods:**

This study included 132 patients with clinically suspected pneumonia at two medical intensive care units of a tertiary care hospital. Bronchoalveolar lavage samples were obtained and divided into three aliquots: one was used for immediate culture, and two, for delayed culture (DC) after storage for 24 hours at 4°C (DC4) and -80°C (DC-80), respectively.

**Results:**

Of 259 bronchoalveolar lavage samples, 84 (32.4%) were positive after immediate culture with 115 relevant culture counts (≥10^4 ^colony-forming units/ml). Reduced (<10^4 ^colony-forming units/ml) or no growth of four and 57 of these isolates was observed in DC4 and DC-80, respectively. The difference between mean bias of immediate culture and DC4 (-0.035; limits of agreement, -0.977 to 0.906) and immediate culture and DC-80 (-1.832; limits of agreement, -4.914 to 1.267) was -1.788 ± 1.682 (*P *< 0.0001). Sensitivity and negative predictive value were 96.5% and 97.8% for DC4 and 50.4% and 75.4% for DC-80, respectively; the differences were statistically significant (*P *< 0.0001).

**Conclusions:**

Bronchoalveolar lavage samples can be processed for culture when stored up to 24 hours at 4°C without loss of diagnostic accuracy. Delayed culturing after storage at -80°C may not be reliable, in particular with regard to Gram-negative bacteria.

## Introduction

Hospital-acquired pneumonia is a serious and frequent infection at the intensive care unit (ICU) [1,2]. Despite advances in prevention and therapy, pneumonia remains a major cause of morbidity and mortality in the ICU [3,4]. Considering the serious prognosis, associated with delayed or inappropriate antimicrobial therapy [5,6] pathogen identification may help to guide treatment and management of pneumonia in the ICU.

Despite the debate of the efficacy of invasive versus noninvasive approaches [7,8], many teams around the world perform distal bronchial sampling for managing pneumonia [9]. Because prior antibiotic treatment may significantly decrease the sensitivity of culture [10], the specimens should ideally be obtained before initiation of treatment. However, in most centers, microbiologic processing is not available around the clock (for example, at night or during weekends), and the appropriate storage of specimens may be of crucial importance to prevent loss of diagnostic accuracy of microbiologic culture.

Freezing and refrigeration of specimens are two laboratory approaches widely used if immediate specimen processing is not available. In a previous study, overnight refrigeration at 4°C of protected specimen brush (PSB) samples did not affect growth of potential pathogens, with the exception of *Haemophilus influenzae *[11]. In a further study, specimens obtained by PSB were stored at -80°C for 24 hours with good reliability of culture, except for *Streptococcus pneumoniae *and *Escherichia coli *[12]. Finally, delayed processing of bronchoalveolar lavage (BAL) and plugged telescopic catheter (PTC) specimens after storage at 4°C for 24 hours was also shown to be an acceptable alternative when immediate microbiologic processing was not feasible [13,14].

Given the scarce data available regarding the optimal storage conditions of respiratory specimens, we aimed to compare refrigeration with freezing, to define the most appropriate storage condition of BAL specimens in clinical practice, when immediate culture is not available.

## Materials and methods

### Patients and inclusion criteria

The study was performed at two medical ICUs (8 beds each) of a tertiary care facility. All patients admitted to the ICUs during the observation period were screened prospectively for clinical suspicion of ventilator-associated pneumonia based on new pulmonary infiltrates on chest radiographs and at least two of the following: fever (>38.5°C) or hypothermia (<36.5°C), leukocytosis (>10 × 10^9^/L) or leukopenia (<3 × 10^9^/L), and purulent tracheal aspirates [15]; patients fulfilling these criteria were included in the study.

Severity of acute illness on intensive care unit admission was assessed by using the Simplified Acute Physiology Score II (SAPS II) [16].

The protocol did not interfere with the clinical routine. BAL via fiberoptic bronchoscopy was requested by the attending physician to investigate the suspicion of pneumonia and to provide guidance for antimicrobial treatment. Thereafter, BAL fluid was divided into three aliquots. One was immediately processed as usual and was used for the clinical decision-making process. The two other aliquots were used to assess the diagnostic accuracy of two storage methods for delayed culture and were not involved in the clinical decision-making process.

The current investigation was performed within the frame of a clinical study, which has been approved by the ethics committee of the Medical University Vienna (Number 303/2007). As our subjects were unable to consent because of sedation and analgesia, the ethics committee waived the need for patient consent.

### Fiberoptic bronchoscopy and bronchoalveolar lavage

A 5.2-mm (outside diameter) fiberoptic bronchoscope (Olympus, BF P 200; Olympus Optical of America, New Hyde Park, NY, USA) was introduced through a special adaptor (catheter mount, Dar-Breathing-System; Tyco Healthcare, Milano, Italy) and advanced close to the lobe at the site of the radiographic signs without suctioning to avoid contamination. Sterile isotonic saline (100 ml) was instilled in 20-ml portions. The first 20 ml was removed to avoid bacterial contamination, whereas the remaining BAL fluid was used for culture. As all ICU patients were already intubated, bronchoscopy was performed via endotracheal tube.

### BAL fluid processing

BAL fluid was immediately brought to the microbiologic laboratory (within 20 minutes after bronchoscopy), where it was separated into three aliquots: one was used for immediate culture (IC), and two, for delayed culture (DC) after storage for 24 hours at 4°C (DC4) and -80°C (DC-80), respectively.

Specimens were processed by using standard routine methods for microscopy, culture, identification, and antimicrobial susceptibility testing (Health Protection Agency: Investigation of bronchoalveolar lavage, sputum and associated specimens. National Standard Method 2008 BSOP 57 Issue 2.2.). Culture was performed semiquantitatively by using a calibrated loop to plate out 10 μl of fluid on each of the used agar media.

All identified microorganisms were recorded. Because microbiologic proof of infection when analyzing BAL is defined as growth of ≥10^4 ^colony-forming units (CFUs)/ml of potential pathogens [17], only those isolates fulfilling these criteria were considered for statistical analysis of the performance characteristics of delayed culture. Thus, no or reduced growth in the delayed processed aliquots, as compared with the corresponding IC aliquots, was recorded and analyzed.

### Statistics

Data are presented as median and 25% to 75% interquartile range (IQR) or mean and standard deviation (SD), as appropriate. Sensitivity, specificity, and predictive values were calculated by using standard formulas and compared by using the McNemar test.

Because the same microorganisms could be detected in different samples of the same patient or different samples of the same bronchoscopy, we calculated robust 95% confidence intervals of the test-performance characteristics to correct for clustering within patients and within BAL samples.

Agreement between immediate and delayed cultures was assessed by the method of Bland and Altman [18], presented as mean bias and limits of agreement (1.96 × SD of the difference). Mean bias of DC4 and DC-80 was compared by using a paired *t *test. Proportions were compared by using the χ^2 ^test. A two-sided *P *value of less than 0.05 was considered statistically significant. Statistical calculations were performed by using Stata 8.0 for Mac (Stata Corp, College Station, TX, USA).

## Results

### Patient characteristics

During a 3-year period 132 patients (46 female and 86 men) with a median age of 62.0 (48.0 to 70.5) years and a median SAPS II on admission of 54.0 (39.3 to 66.0) were included in the study. In 105 patients, one bronchoscopy was performed, from which 166 samples were obtained. In 23 patients, two bronchoscopies, in two patients, three bronchoscopies, and in two further patients, four bronchoscopies were performed, from which 72 samples, eight samples, and 13 samples were obtained, respectively. On average, 1.3 (± 0.6) bronchoscopies were performed, and 2.0 (± 1.1) samples were obtained per patient. In 70 patients, the bronchoscopies were performed bilaterally. All patients were mechanically ventilated. Median overall length of mechanical ventilation (MV) and median length of MV before bronchoscopy were 11 (7 to 20) and 5 (1 **to **11) days, respectively. Median ICU length of stay was 17 (9 to 31) days, and ICU mortality was 28.2%.

### Immediate culture

Of a total of 259 BAL samples, growth was observed in 216 (83.4%), and significant growth (≥10^4 ^CFU/ml) of at least one potential pathogen in 84 (32.4%); the latter originated from 55 patients. Overall, 115 potential pathogens yielded relevant counts (≥10^4 ^CFU/ml); numbers of potential pathogens recovered per specimen type are shown in Table 1. Of these 115 microorganisms, 18 different bacterial species were identified; 15 species of Gram-negative bacteria, accounting for 74 isolates, and three species of Gram-positive bacteria, accounting for 41 isolates. All microorganisms cultured from immediately processed specimens are shown in Table 2. Of the 115 potential pathogens, 56 were isolated from 32 specimens originating from 17 patients, of a total of 39 patients having not received any antimicrobial treatment for at least 72 hours before bronchoscopy; 59 were isolated from 52 specimens originating from 38 patients of a total of 106 patients having received antimicrobial treatment for at least 24 hours (median 96, 48 to 192 hours) before bronchoscopy. BAL specimens before and after antimicrobial therapy were obtained from 13 patients, who were therefore counted on both the untreated and the treated groups of patients. The ratio of the number of potential pathogens to the total number of patients was statistically significantly higher in the untreated than in the treated group (56 of 39 versus 59of 106; OR, 2.6; 95% CI, 1.5 to 4.5; *P *= 0.0004); however, this did not apply for the ratio of the number of corresponding patients to the total number of patients in each group (17of 39 versus 38 of 106; OR, 1.2; 95% CI, 0.6 to 2.5; *P *= 0.599). Furthermore, the ratio of samples with significant growth to the number of specimens examined was 32 (42.1%) of 76 in untreated and 52 (28.4%) of 183 in treated patients (OR, 1.5; 95% CI, 0.9 to 2.6; *P *= 0.139).

**Table 1:** Specimen type and number of potential pathogens (≥10^4 ^CFU/ml)

No. potential pathogens	IC	DC4	DC-80
0	175	177	221
1	61	61	23
2	15	13	10
3	8	8	5

Total	259	259	259

IC, immediate culture; DC4, delayed culture after storage at 4°C; DC-80, delayed culture after storage at -80°C.

**Table 2:** Significant growth (≥10^4 ^CFU/ml) of potential pathogens recovered from IC, DC4, and DC-80

Species of potential pathogens	IC	DC4	Mean decrease ± SD	DC-80	Mean Decrease ± SD
**Gram-positive bacteria**					
*Staphylococcus aureus*	21	20	0.190 ± 0.510	19	0.238 ± 0.590
*Enterococcus faecalis *	15	15	0	13	0.400 ± 0.910
*Streptococcus pneumoniae*	5	5	0	4	0.600 ± 0.890
**Gram-negative bacteria**					
*Pseudomonas aeruginosa*	28	28	-0.071 ± 0.460	3	3.250 ± 1.290
*Escherichia coli*	11	9	0.182 ± 0.400	6	2.180 ± 1.080
*Haemophilus influenzae*	8	8	0	8	1.750 ± 0.710
*Enterobacter cloacae *	7	7	0.143 ± 0.690	3	2.000 ± 1.000
*Klebsiella oxytoca*	3	3	0	0	-
*Klebsiella pneumoniae*	3	3	0	0	3.000 ± 1.000
*Stenotrophomonas maltophilia*	3	3	0.333 ± 0.580	1	2.000 ± 1.000
*Hafnia alvei*	2	2	-0.500 ± 0.710	0	1.000 ± 0.000
*Morganella morganii *	2	2	0	0	2.000 ± 1.000
*Acinetobacter baumannii*	2	2	0	1	0.500 ± 0.710
*Citrobacter koseri*	1	1	-	0	-
*Enterobacter aerogenes*	1	1	-	0	-
*Pantoea *spp.	1	0	-	0	-
*Providencia alcalifaciens*	1	1	-	0	-
*Serratia marcescens*	1	1	-	0	-

Total	115	111		58	

IC, immediate culture; DC4, delayed culture after storage at 4°C; DC-80, delayed culture after storage at -80°C. Mean decrease expressed as log_10 _of CFU/ml ± SD

### Delayed culture

**Figure 1:**
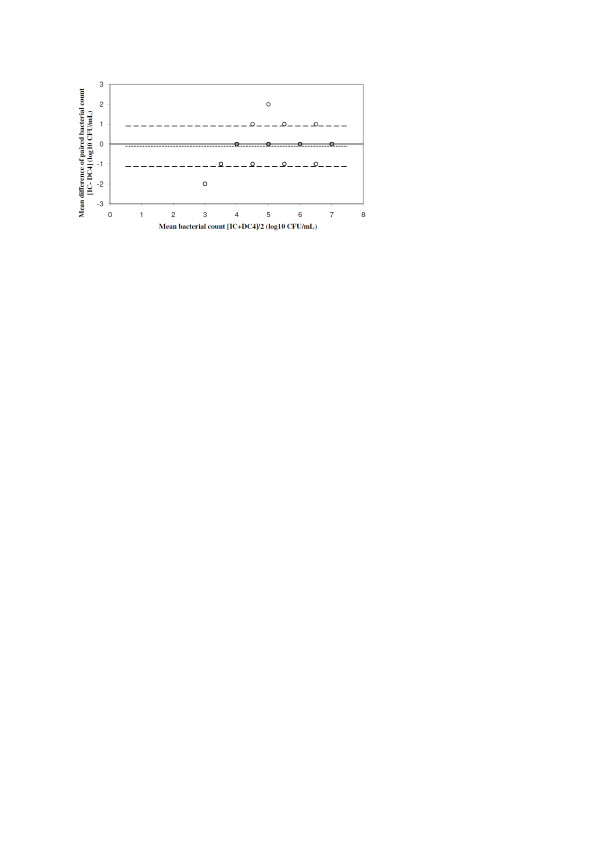
**Agreement of bacterial counts from paired IC and DC4 of all identified species (*n *= 115)**. Mean bias (dotted line), limits of agreement (1.96*SD of the difference, dashed line). IC, immediate culture; DC4, delayed culture after storage at 4°C.

Considering 10^4 ^CFU/ml as the threshold for significant pathogen growth, IC and DC4 paired samples were concordant in 257 (99.2%) of 259 cases; thus, the counts of four potential pathogens (cultured from two samples, two pathogens each; Table 1) decreased below 10^4 ^CFU/ml on storage at 4°C. The decrease of cfu counts in DC4 as compared with IC is shown in Table 2 for each of the identified species. The mean decrease of Gram-positive and Gram-negative bacteria was 0.098 ± 0.374 log_10 _and 0.000 ± 0.523 log_10_, respectively. The performance characteristics of DC4 as compared to IC are shown in Table 3. The agreement between cfu counts of IC and DC4 is shown in Figure 1. Mean bias was -0.035, and limits of agreement were -0.977 to 0.906.

**Table 3:** Performance characteristics of DC4 and DC-80 in comparison to IC

	DC4 (95% CI)	DC-80 (95% CI)	*P *value
Sensitivity	96.5% (90.8-98.9)	50.4% (41.0-59.8)	<0.00001
Specificity	100% (97.3-100)	100% (97.3-100)	0.99
Positive predictive value	100% (95.8-100)	100% (92.3-100)	0.99
Negative predictive value	97.8% (94.0-99.3)	75.4% (69.3-80.7)	<0.0001

DC4, delayed culture after storage at 4°C; DC-80, delayed culture after storage at -80°C; IC, immediate culture; CI, confidence interval.

**Figure 2:**
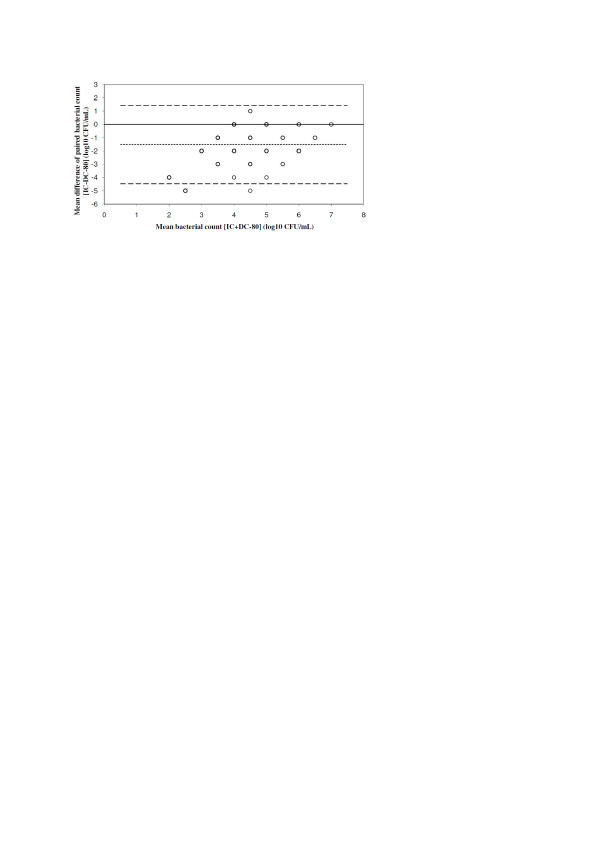
**Agreement of bacterial counts from paired IC and DC-80 of all identified species (*n *= 115)**. Mean bias (dotted line), limits of agreement (1.96*SD of the difference, dashed line). IC, immediate culture; DC-80, delayed culture after storage at -80°C.

IC and DC-80 paired samples were concordant in 213 (82.2%) of 259 cases with respect to the threshold of 10^4 ^CFU/ml for significant pathogen growth; the counts of 57 potential pathogens (cultured from a total of 46 samples; Table 1) decreased below 10^4 ^CFU/ml on storage at -80°C. In fact, 16 Gram-negative bacteria, of which eight were *P. aeruginosa*, did not grow at all. The decrease of cfu counts in DC-80 as compared with IC is shown in Table 2 for each of the identified species. The mean decrease of Gram-positive and Gram-negative bacteria was 0.342 ± 0.728 log_10 _and 2.595 ± 1.334 log_10_, respectively. The performance characteristics of DC-80 in comparison to IC are shown in Table 3. The agreement between cfu counts of IC and DC-80 is shown in Figure 2. Mean bias was -1.832, and limits of agreement were -4.917 to 1.267.

The difference between mean bias of IC versus DC4 and IC versus DC-80 was -1.788 ± 1.682 (*P *< 0.0001). In six BAL samples, cfu counts in DC4 exceeded by one log_10 _those found in IC. However, in none of the 175 samples, which did not show any significant growth of potential pathogens, the count of any of the pathogens detected was ≥10^4 ^CFU/ml on storage at 4°C or -80°C. Samples showing no growth in IC were sterile also in both DC4 and DC-80.

Prior antimicrobial treatment did not affect growth of potential pathogens in relevant counts in DC4; thus, two of 56 and two of 59 potential pathogens isolated from untreated and treated patients, respectively, showed reduction in growth below 10^4 ^CFU/ml. In DC-80, however, antimicrobial treatment was significantly associated with failure to detect relevant counts of potential pathogens; the respective numbers were 20 of 56 and 37 of 59 (*P *= 0.004).

Because the same microorganisms could be detected in different samples of the same patient or different samples from the same bronchoscopy, we calculated robust 95% confidence intervals of the test-performance characteristics to correct for clustering within patients and within BAL samples. Correction for clustering within patients and BAL samples did not affect the performance characteristics of DC4 and DC-80 (data not shown), which were shown to differ statistically significantly (*P *< 0.0001) with regard to sensitivity and negative predictive value (Table 3).

## Discussion

In the present study, the influence of storage conditions on growth of pathogens potentially causing pneumonia in ICU patients was investigated in a large number of BAL specimens. Microbiologic culture was highly sensitive on storage of BAL at 4°C for up to 24 hours; however, a considerable loss of sensitivity was observed after storage at -80°C.

Although the benefit of detecting the causative organism in ventilator-associated pneumonia (VAP) is undisputed, the approach by which this can be achieved is under debate. The impact of microbiologic findings of distal bronchial sampling by BAL on mortality of ICU patients has been discussed controversially [7,8,19]. Recent trials failed to prove the advantage of BAL on outcome and antibiotic use in comparison with other less invasive or noninvasive diagnostic approaches [8,20-22]. Conversely, it has been shown that, compared with a noninvasive management strategy, an invasive management strategy is significantly associated with fewer deaths at 14 days, earlier attenuation of organ dysfunction, and less antibiotic use in patients suspected of having VAP [19]. Furthermore, invasive diagnostic testing may increase the physician's confidence in diagnosis and management of VAP and may allow greater ability to limit or discontinue antibiotic treatment [23]. However, BAL is considered a reliable method for establishing a definite etiologic diagnosis of pneumonia, and ICU teams will continue to use BAL for managing VAP and deriving clinical decisions. However, the clinical decision-making process, and consequently the outcome for patients with VAP, will depend on the quality of the clinical specimen, which may be affected to a considerable extent by inappropriate storage conditions.

In comparison with immediate microbiologic culture, our results demonstrate an excellent test performance after storage of BAL specimens at 4°C for up to 24 hours, but a considerable decrease of bacterial growth on storage at -80°C. This effect was more pronounced in the Gram-negative bacterial spectrum and resulted in a highly significant reduction of culture sensitivity and negative predictive value. This is of importance because the prognosis for aerobic, Gram-negative bacilli in VAP is considerably worse than that for infection with Gram-positive pathogens, even when microorganisms are fully susceptible to antibiotics [24]. Thus, the loss of diagnostic performance for Gram-negative pathogens after storage of BAL at -80°C may lead to an underestimation of a particularly serious clinical condition. The highest reduction was observed in the bacterial counts of *Pseudomonas aeruginosa *isolates (3.25 ± 1.29 log_10_). Furthermore, of the 28 isolates of this pathogen grown in relevant counts during routine immediate culture, only three exceeded 10^4 ^CFU/ml in samples stored at -80°C before culture. This is of particular importance, because in nearly all studies, *P. aeruginosa *is the most frequently isolated bacterium in VAP, and episodes caused by the pathogen are associated with a significantly higher mortality than are those caused by other microorganisms [25-29]. In a study by Fagon *et al*. [27], after matching for the severity of underlying disease and various other important variables, the attributable mortality was 42.8%, and the risk ratio for death was 2.5 for pneumonia due to *P. aeruginosa *or *Acinetobacter *species. Thus, early pathogen identification and initiation of appropriate therapy is crucial in *Pseudomonas *pneumonia. Therefore, storage of BAL at -80°C resulting in impaired growth of *P. aeruginosa *and other pathogens of VAP should be considered inappropriate in clinical practice.

In the present study, prior antimicrobial treatment was associated in a statistically significant manner with failure to detect sufficient counts of potential pathogens after storage at -80°C. Thus, microorganisms may be more susceptible to freezing/thawing on exposure to antimicrobials, and treatment may be one of the reasons for the considerably lower sensitivity of culture subsequent to this procedure.

In the study by Georges *et al*. [12], growth of *P. aeruginosa *was only moderately affected by storage of PSB specimens at -80°C, and none of the isolates failed to grow in clinically relevant counts (≥10^3 ^CFU/ml) after sample freezing. In this previous study, a mean overall decrease of bacterial counts of 1.00 ± 1.44 log_10 _was found, and the authors concluded that PSB specimens can be frozen at -80°C with good diagnostic reliability of culture, except for *Streptococcus pneumoniae *and *Escherichia coli*. The impact of sample freezing on growth of *E. coli *but not of *S. pneumoniae *is in accordance with the data of the present study. Apparent discrepancies between the previous and the current study may be due mainly to the different consistency and nature of PSB as compared with BAL and the rather small number of samples analyzed by Georges *et al*. [12].

In contrast to the significant impact of BAL specimen freezing on growth of Gram-negative bacteria, refrigeration at 4°C for 24 hours was shown to affect the culture of these microorganisms to only a minor extent. This finding is in accordance with data presented in previous smaller studies, suggesting that 24 hours of refrigeration of PTC and BAL specimens does not result in loss of the diagnostic accuracy of culture [13,14,30]. Immediate storage and rapid processing may have had an impact on the good performance of culture in specimens stored at 4°C in this and previous studies. However, because refrigeration is available and easily practicable in most intensive care units, BAL sampling could be performed around the clock with the certainty that this will not affect the diagnostic accuracy of subsequent culture.

In a study by Forceville *et al*. [30], storage of PSB and BAL specimens for 48 hours at 4°C revealed an acceptable reproducibility of culture, except for *Haemophilus influenzae*. The authors state that a 1-log reduction of the cut-off for significant growth has been associated with a better performance of culture subsequent to storage of specimens. In the present study, the use of ≥10^3 ^CFU/ml as threshold did not affect the performance of DC4. In contrast, the sensitivity and negative predictive value of DC-80 increased to 73.9% and 85.4%, respectively, but overall values remained considerably lower in comparison to DC4.

However, the fact that the effect of longer periods of refrigeration has not been investigated may be a limitation of the present study, because this may be of particular importance in facilities where specimen processing (for example, during the weekend) is not feasible. Furthermore, although storage conditions evaluated here and especially refrigeration are most commonly used, it may be of importance to elucidate whether, for example, more-gentle freezing/thawing conditions may be also appropriate for the storage of BAL specimens. Finally, sample quality in different storage conditions and its influence on culture results was not evaluated and may represent a further limitation of the present study.

## Conclusions

Our study clearly demonstrates that storage of BAL specimens at 4°C for up to 24 hours allows the accurate detection of VAP pathogens by culture. Therefore, even if microbiologic processing may not be available 24 hours per day, BAL sampling can be performed around the clock without loss of diagnostic accuracy. Storage at -80°C inhibits growth of Gram-negative bacteria to a considerable extent and therefore cannot be recommended for clinical use.

## Key messages

• Bronchoalveolar lavage samples can be processed for culture when stored up to 24 hours at 4°C without loss of diagnostic accuracy.

• Storage at -80°C inhibits growth of Gram-negative bacteria to a considerable extent and therefore cannot be recommended for clinical use.

## Abbreviations

BAL: bronchoalveolar lavage; CFU: colony-forming unit; DC4: delayed culture after storage for 24 hours at 4°C; DC-80: delayed culture after storage for 24 hours at 4°C to 80°C; ICU: intensive care unit; PSB: protected specimen brush; PTC: plugged telescopic catheter; VAP: ventilator-associated pneumonia.

## Competing interests

The authors declare that they have no competing interests.

## Authors' contributions

NK, AM, and PS assisted in conception and design. VF, JW, PS, and AB performed acquisition of data and data analyses; HH, statistical and data analyses, and statistical advisor for the study and assistance with significant manuscript revision. NK, AM, and PS participated in analysis and interpretation of data and drafting of the manuscript. CM and AH aided in interpretation of data and critical revision of the manuscript for important intellectual content. All authors read and approved the final manuscript for publication.
